# Supplementary material for: RADIX: a deep learning framework that maps root barriers across species and reveals genetic and environmental contributions
Source: bioRxiv. 2026 Aug 10:2026.08.07.743584. Preprint. [Version 1] doi: 10.64898/2026.08.07.743584 (PMC13640580; doi:10.64898/2026.08.07.743584)
Supplement: Supplementary file 1 [file NIHPP2026.08.07.743584v1-supplement-1.pdf]

bioRxiv preprint doi: https://doi.org/10.64898/2026.08.07.743584; this version posted August 10, 2026. The copyright holder for this preprint (which was not certified by peer review) is the author/funder, who has granted bioRxiv a license to display the preprint in perpetuity. It is made available under a CC-BY 4.0 International license.

Supplementary Figures

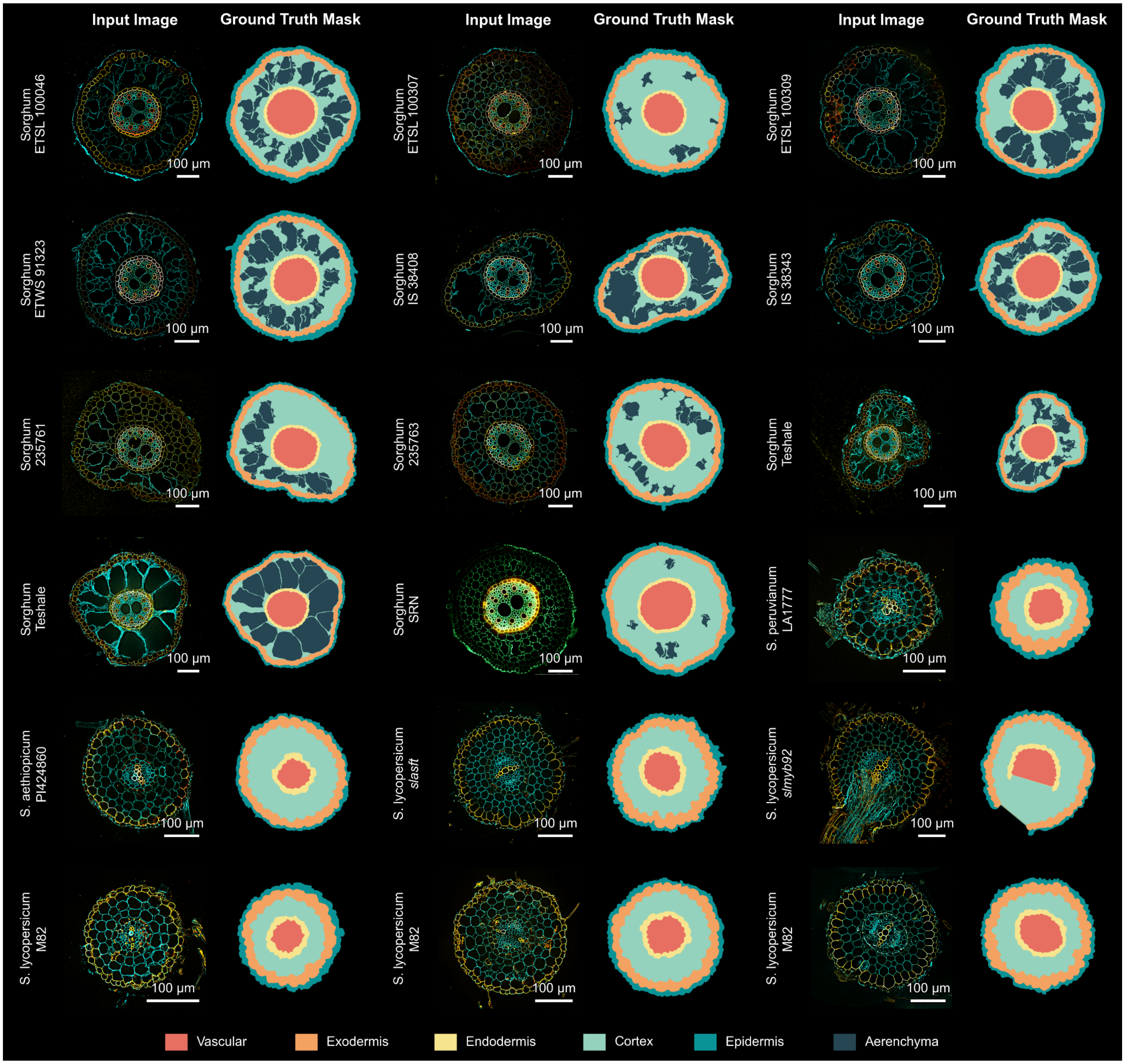

Supplementary Figure 1. *Additional representative samples in the training data set in three channel composites (DAPI in cyan, FITC in yellow, and TRITC in red) with corresponding ground truth annotation used during supervised training.*

bioRxiv preprint doi: https://doi.org/10.64898/2026.08.07.743584; this version posted August 10, 2026. The copyright holder for this preprint (which was not certified by peer review) is the author/funder, who has granted bioRxiv a license to display the preprint in perpetuity. It is made available under a CC-BY 4.0 International license.

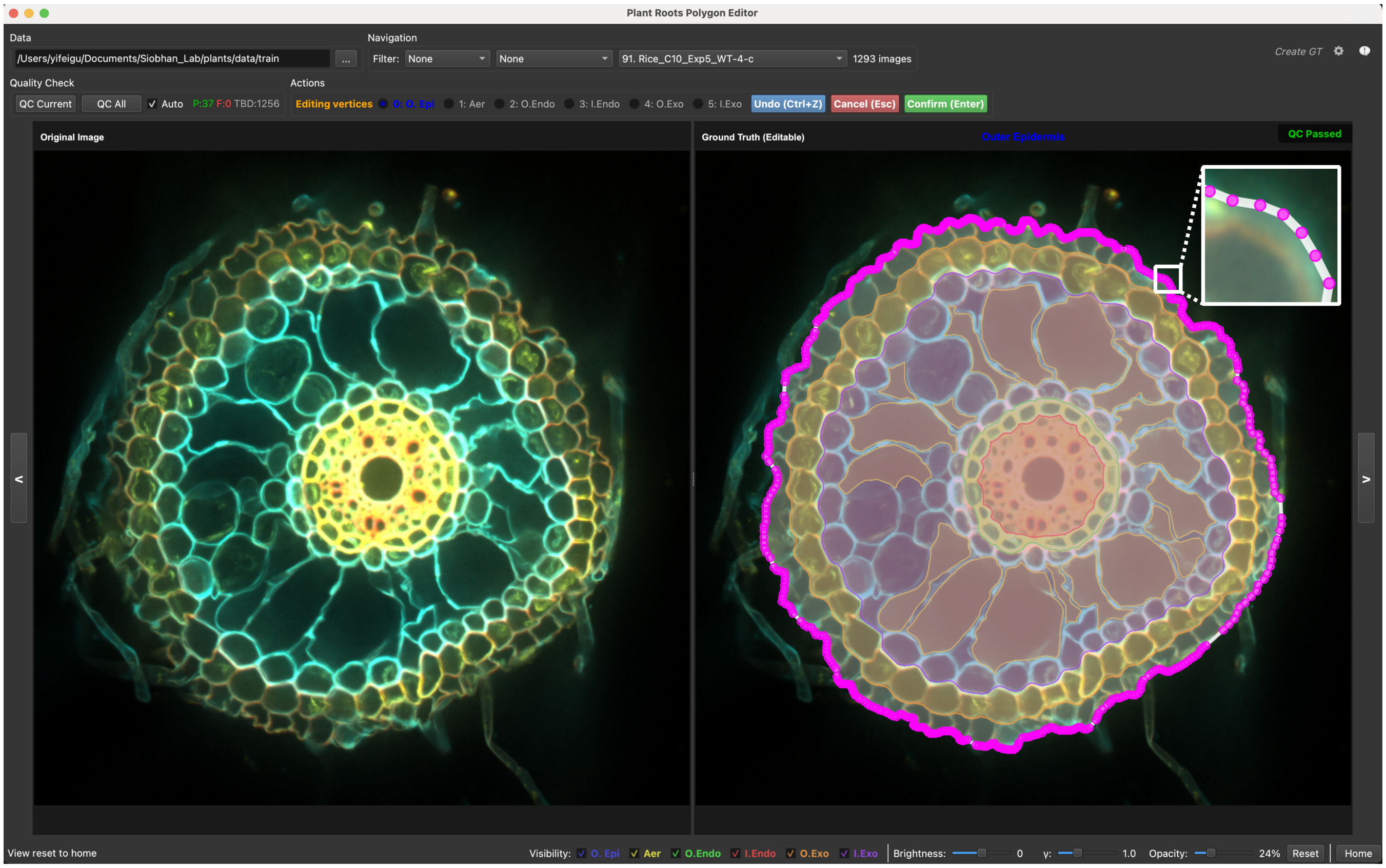

Supplementary Figure 2. Annotation review tool. *A screenshot of the GUI built for rapid annotation review.*

bioRxiv preprint doi: https://doi.org/10.64898/2026.08.07.743584; this version posted August 10, 2026. The copyright holder for this preprint (which was not certified by peer review) is the author/funder, who has granted bioRxiv a license to display the preprint in perpetuity. It is made available under a CC-BY 4.0 International license.

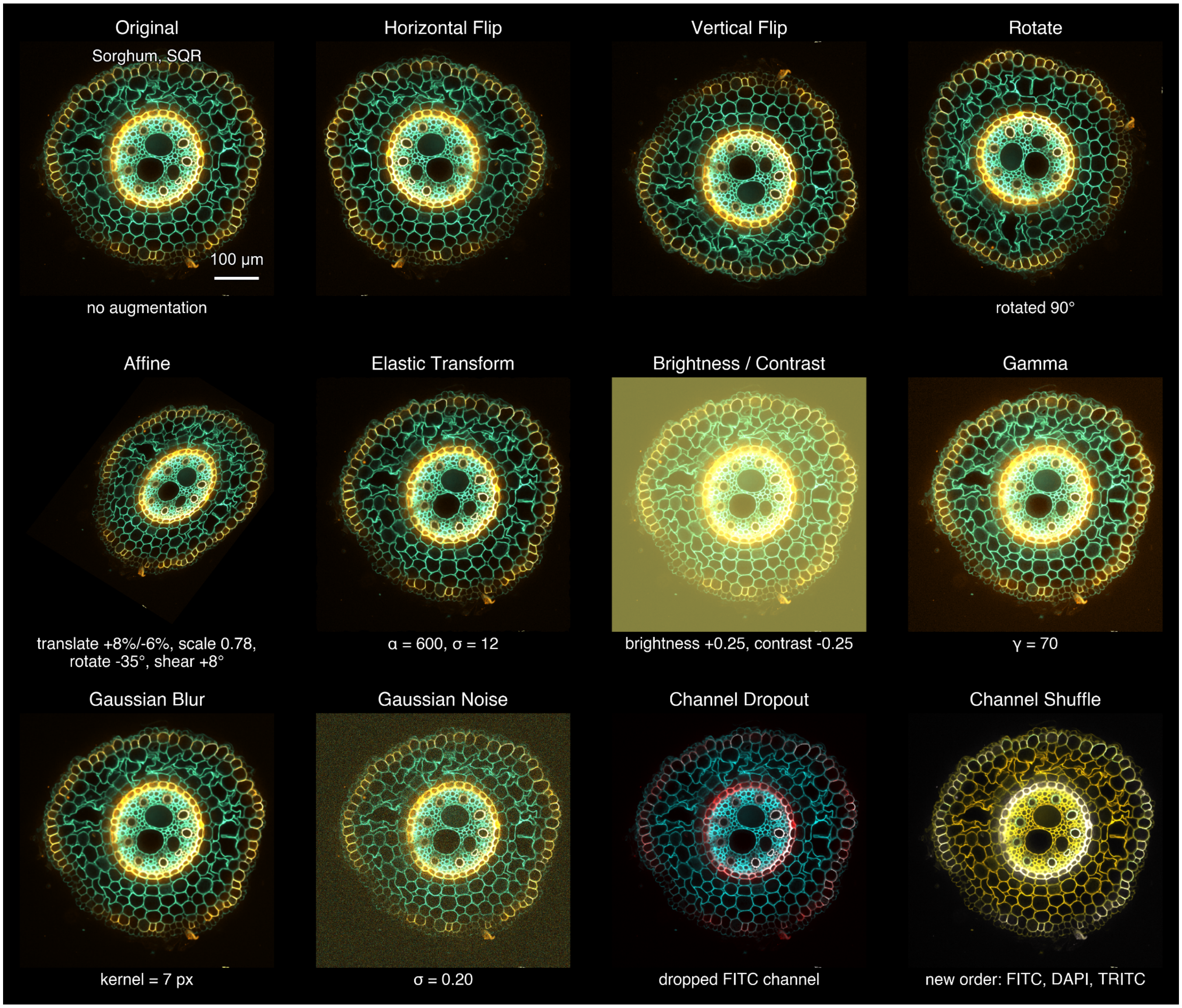

Supplementary Figure 3. An example image is shown with different types of augmentation operation used during training. *Multiple of these operations could be applied with random parameters described in Methods during training. The top-left panel is the unaltered sample for reference; the remaining eleven panels show, in order, 90° random rotation, horizontal flip, vertical flip, affine transformation (translation, isotropic scaling, in-plane rotation, and shear), elastic deformation, brightness and contrast jitter, gamma adjustment, Gaussian blur, additive Gaussian noise, channel dropout (one of the three fluorescence channels set to zero), and channel shuffle (the three channels randomly permuted). The exact parameter values used in this figure are annotated below the corresponding image. All channels are displayed using the paper convention: DAPI in cyan, FITC in yellow, and TRITC in red, additively blended. Geometric operations (90° rotation, flips, affine, elastic) are applied identically to the image and to the semantic segmentation mask during training; Intensity and channel operations modify only the image. During training, each operation is sampled independently at the probabilities reported in the Methods section.*

bioRxiv preprint doi: https://doi.org/10.64898/2026.08.07.743584; this version posted August 10, 2026. The copyright holder for this preprint (which was not certified by peer review) is the author/funder, who has granted bioRxiv a license to display the preprint in perpetuity. It is made available under a CC-BY 4.0 International license.

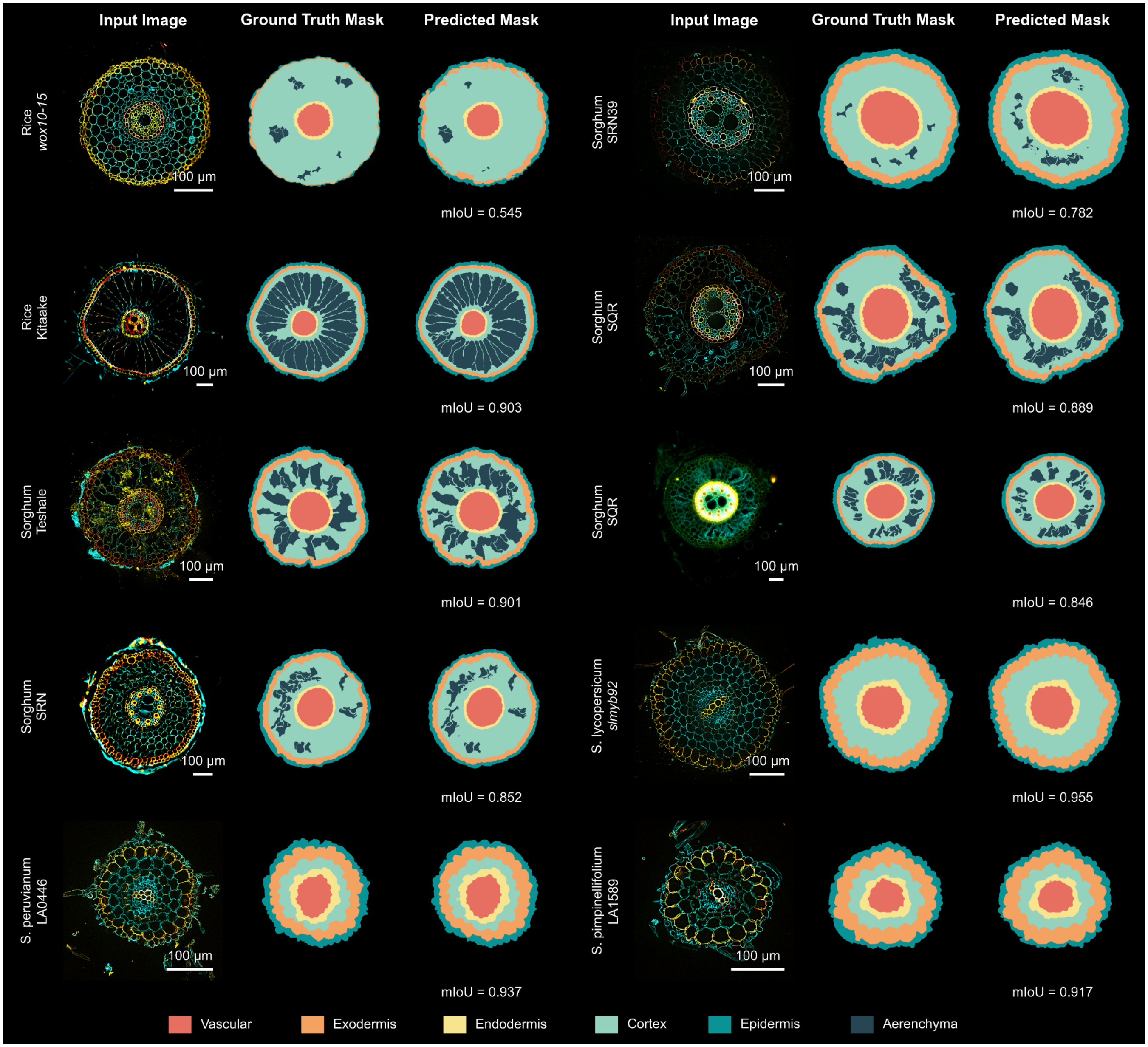

Supplementary Figure 4. *Additional representative examples of model predicted mask vs. ground truth mask from test data sets.*

bioRxiv preprint doi: https://doi.org/10.64898/2026.08.07.743584; this version posted August 10, 2026. The copyright holder for this preprint (which was not certified by peer review) is the author/funder, who has granted bioRxiv a license to display the preprint in perpetuity. It is made available under a CC-BY 4.0 International license.

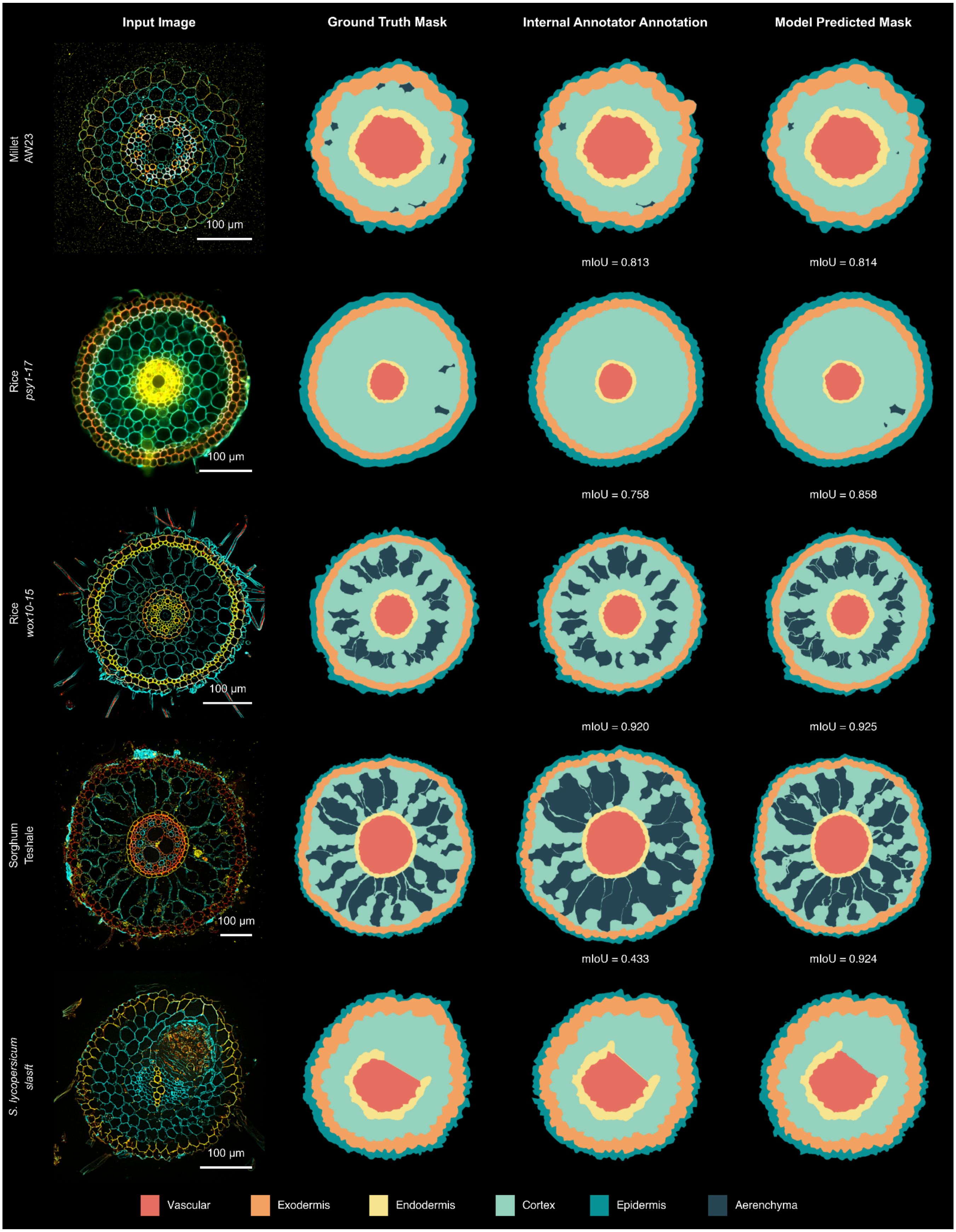

Supplementary Figure 5. Visual comparison of ground-truth, internal-annotator annotations, and RADIX predictions on five representative test-set samples. *Each row shows one root cross-section. Column from left to right: (1) the input fluorescence composite (DAPI in cyan, FITC in yellow, TRITC in red); (2) the consensus ground-truth annotation used*

bioRxiv preprint doi: https://doi.org/10.64898/2026.08.07.743584; this version posted August 10, 2026. The copyright holder for this preprint (which was not certified by peer review) is the author/funder, who has granted bioRxiv a license to display the preprint in perpetuity. It is made available under a CC-BY 4.0 International license.

*during training; (3) an independent re-annotation by a second expert plant biologist, blinded to the consensus ground truth, drawn from the balanced subset of sixteen test-set samples to estimate inter-annotator agreement (**Fig. 2d**); (4) the prediction of RADIX. Masks are colored by anatomical class using the palette shown in the bottom legend (epidermis, exodermis, cortex, aerenchyma, endodermis, stele). The five samples span both monocots (millet, rice, sorghum) and a dicot (Solanum lycopersicum), and illustrate the typical pattern observed across all sixteen re-annotated samples(**Sup. Table 5**).*

bioRxiv preprint doi: https://doi.org/10.64898/2026.08.07.743584; this version posted August 10, 2026. The copyright holder for this preprint (which was not certified by peer review) is the author/funder, who has granted bioRxiv a license to display the preprint in perpetuity. It is made available under a CC-BY 4.0 International license.

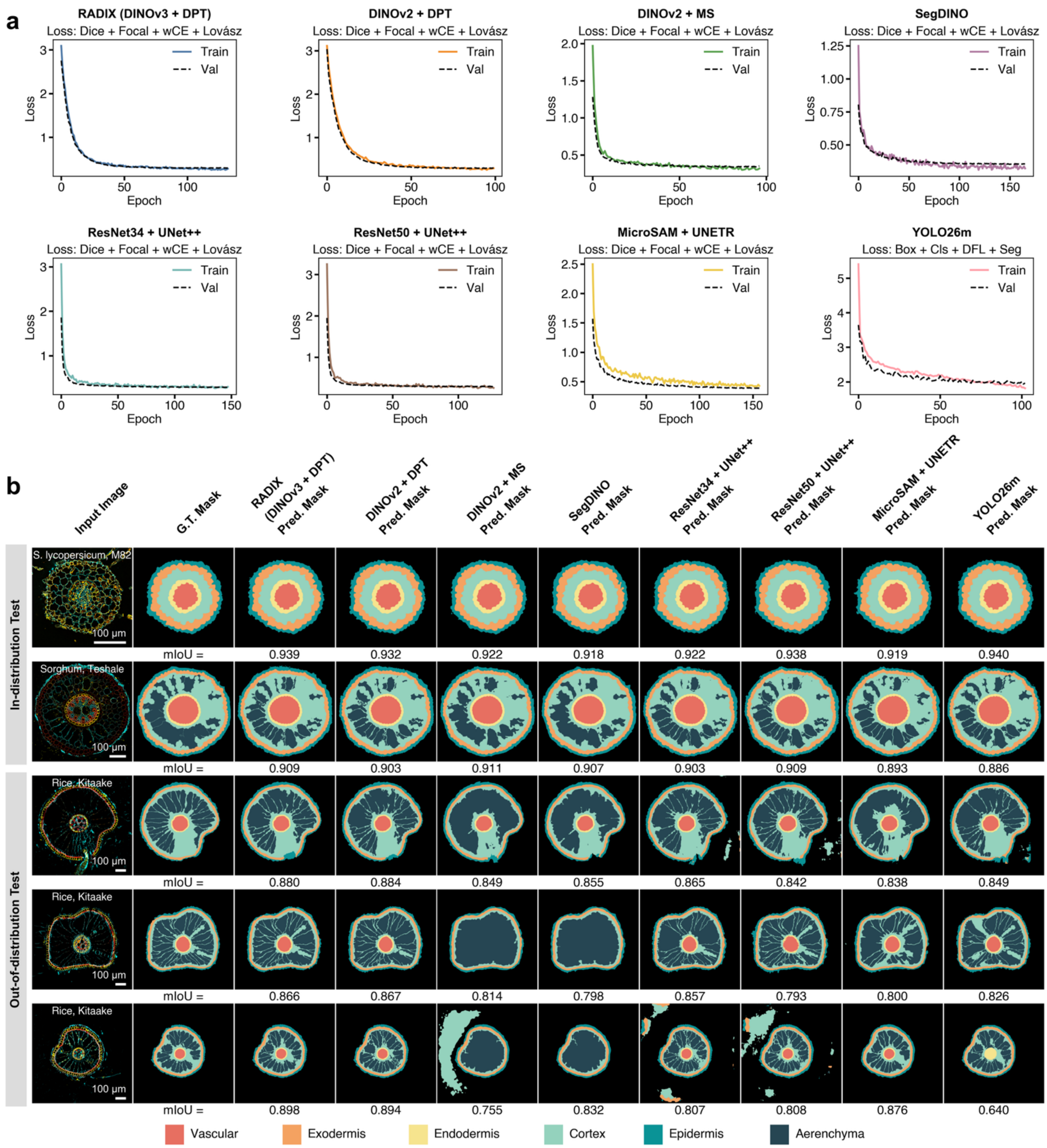

Supplementary Figure 6. Architecture comparison of eight segmentation models. *(a) Training and validation loss curves for each model, with the loss formulation it was trained on annotated in the subplot title. All seven semantic models (RADIX = DINOv3-S/16 + DPT decoder; ResNet34 + UNet++; ResNet50 + UNet++; DINOv2-S/14 with the same DPT decoder; DINOv2 with a multi-scale linear head; SegDINO; MicroSAM ViT-B + UNETR) optimize the same Dice + Focal + cross-entropy (wCE) + Lovász combination at 1024x1024 resolution. YOLO26m-seg, an instance-segmentation model, is trained with the Ultralytics box + class + DFL + segmentation loss. Curves are recorded per epoch from the corresponding training log; training loss is shown in color and validation loss as a dashed black line. All eight models reach a low, stable plateau within the 200-epoch training budget (early stopping triggered when validation loss failed to improve for 15 consecutive epochs), indicating that the*

bioRxiv preprint doi: https://doi.org/10.64898/2026.08.07.743584; this version posted August 10, 2026. The copyright holder for this preprint (which was not certified by peer review) is the author/funder, who has granted bioRxiv a license to display the preprint in perpetuity. It is made available under a CC-BY 4.0 International license.

chosen budget is sufficient for convergence and that the comparison in panel b is not confounded by under-training. (b) Per-sample predictions on five examples: two in-distribution test images and three out-of-distribution Zeiss images that no model saw during training or validation. Columns from left to right show the three channels composite (DAPI in cyan, FITC in yellow, TRITC in red), the ground-truth mask, and the prediction of each model painted in the anatomy palette shown in the legend. The mean IoU printed below each prediction is averaged across the six anatomical classes. RADIX matches or exceeds every alternative on the two in-distribution samples and is the most robust model under the Zeiss out-of-distribution shift, while other architectures show characteristic failure modes there. DINOv2-MS-Linear incorrectly predicts exodermis on the Solanum M82 sample, ResNet50 + UNet++ produces a fragmented epidermis on one of the Zeiss rice samples, and YOLO26m-seg collapses the central root region on the Solanum M82 sample.

bioRxiv preprint doi: https://doi.org/10.64898/2026.08.07.743584; this version posted August 10, 2026. The copyright holder for this preprint (which was not certified by peer review) is the author/funder, who has granted bioRxiv a license to display the preprint in perpetuity. It is made available under a CC-BY 4.0 International license.

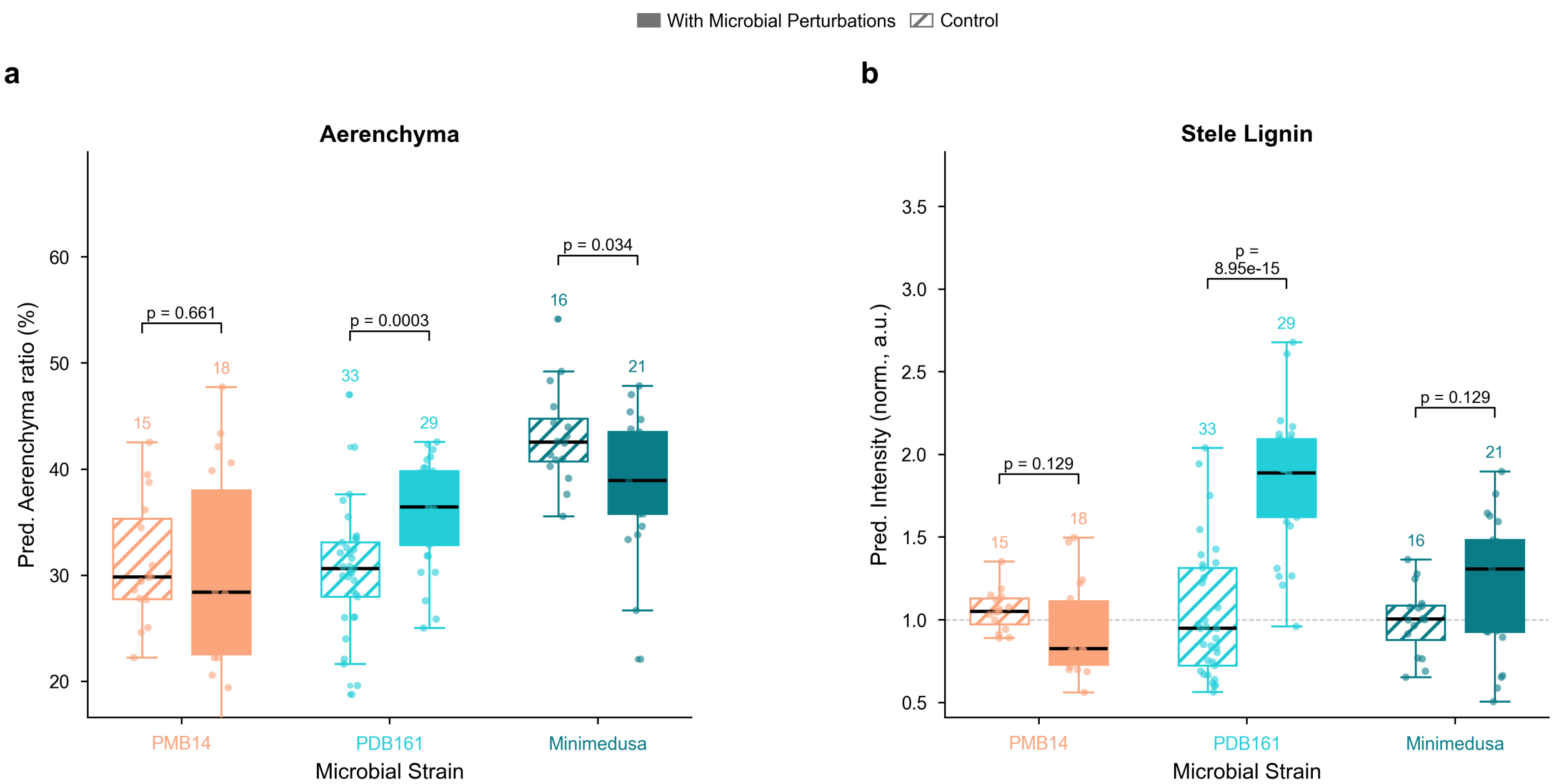

Supplementary Figure 7. *Effects of microbial inoculants on sorghum root barrier traits: aerenchyma and stele lignin. Boxplots showing aerenchyma and stele lignin measurements in 7-day-old sorghum roots treated with three microbial isolates featured in Figure 5b, each compared to within-experiment controls. a, Aerenchyma ratio (cross-sectional area of air spaces relative to total root area, %). b, Stele (vascular) TRITC intensity (lignin proxy), normalized to the per-experiment control mean (dashed line at 1.0). These two traits are not shown for these isolates in Figure 5b and are reported here separately. Isolates: PMB14 (salmon, Exp. 1), PDB161 (cyan, Exp. 4), Minimedusa (teal, Exp. 9). Within-experiment controls are shown as hatched boxes to the left of each treatment group. Numbers above boxes indicate total cross-sections per group. Boxes span the interquartile range (IQR); center lines indicate medians; whiskers extend to the most extreme value within 1.5xIQR; individual data points are overlaid. P-values shown above brackets are BH-adjusted; statistical analysis follows the same approach as for Figure 5b (linear mixed-effects model with Experiment as random effect, Benjamini-Hochberg FDR correction across all traits and treatments), as described in detail in the* ***Methods****.*

bioRxiv preprint doi: https://doi.org/10.64898/2026.08.07.743584; this version posted August 10, 2026. The copyright holder for this preprint (which was not certified by peer review) is the author/funder, who has granted bioRxiv a license to display the preprint in perpetuity. It is made available under a CC-BY 4.0 International license.

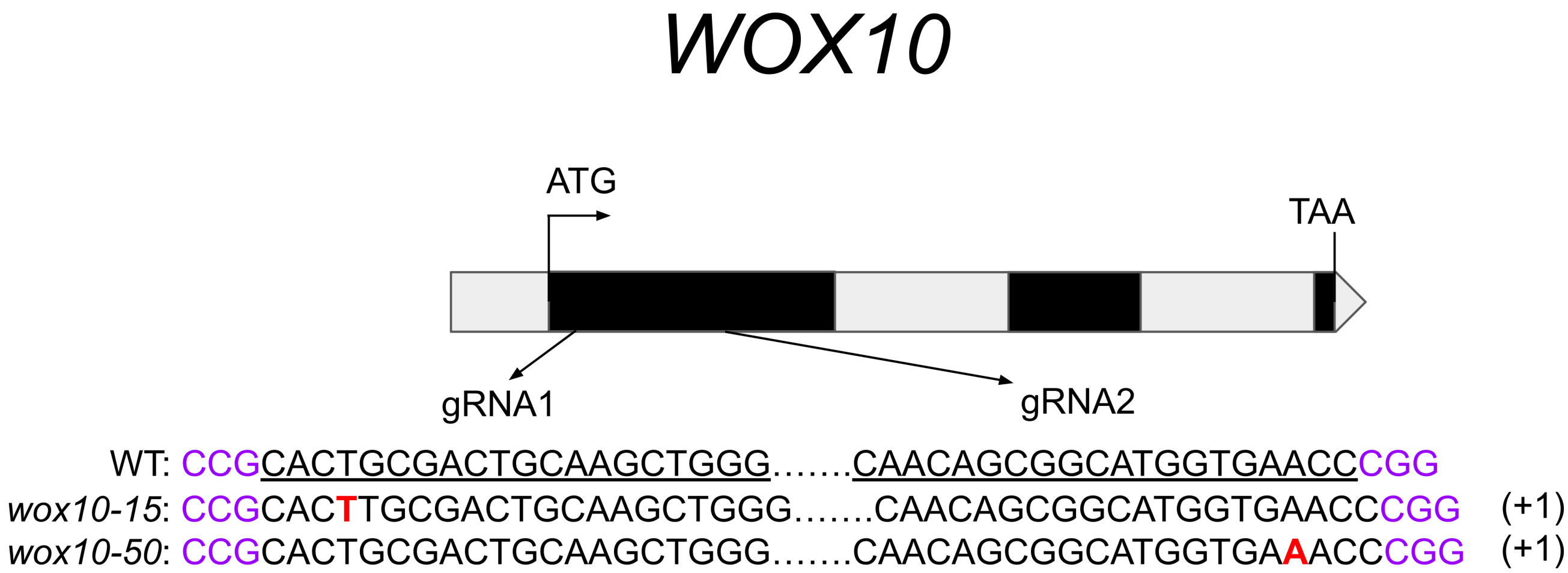

Supplementary Figure 8. CRISPR-Cas9-mediated disruption of *WOX10* in *Oryza sativa*. *Schematic of the WOX10 (Os08g0242400) gene structure showing exons (black boxes) and introns (white boxes). ATG and TAA indicate the start and stop codons, respectively. Arrows indicate the positions of two guide RNAs (gRNA1 and gRNA2). Underlined sequences show the gRNA target sites; purple indicates the PAM sequence (CGG). The sequences of the wild-type (WT) and two independent CRISPR-Cas9 wox10 mutant alleles (wox10-15 and wox10-50) are shown below. Red nucleotides indicate the 1-bp insertions (+1) predicted to cause a frameshift mutation in each allele.*

bioRxiv preprint doi: https://doi.org/10.64898/2026.08.07.743584; this version posted August 10, 2026. The copyright holder for this preprint (which was not certified by peer review) is the author/funder, who has granted bioRxiv a license to display the preprint in perpetuity. It is made available under a CC-BY 4.0 International license.

Supplementary Tables

Supplementary Table 1. Test sets diversity by species and genotype (n= 185 in-distribution, 35 out-of-distribution).

Species	Genotype	In-distribution Test	Out-of-distribution Test	Total
Millet	AW23	32	0	32
Rice	Kitaake	9	35	44
	psy1-17	3	0	3
	psy1-9	3	0	3
	wox10-15	42	0	42
Sorghum	SQR	8	0	8
	SRN	5	0	5
	SRN39	4	0	4
	Teshale	42	0	42
Solanum lycopersicum	M82	22	0	22
	slasft	4	0	4
	slmyb92	5	0	5
Solanum abutiloides	Baker_Creek	1	0	1
Solanum candidum	994750097	1	0	1
Solanum cheesmaniae	LA1407	1	0	1
Solanum insanum	INS1	1	0	1
Solanum peruvianum	LA0446	1	0	1
Solanum pimpinellifolium	LA1589	1	0	1

bioRxiv preprint doi: https://doi.org/10.64898/2026.08.07.743584; this version posted August 10, 2026. The copyright holder for this preprint (which was not certified by peer review) is the author/funder, who has granted bioRxiv a license to display the preprint in perpetuity. It is made available under a CC-BY 4.0 International license.

Supplementary Table 2. Per-class IoU of RADIX on the in-distribution and out-of-distribution test sets.

Anatomical Class	In-distribution Test IoU (mean ± s.d., n = 185)	Out-of-distribution Test IoU (mean ± s.d., n = 35)
Vascular	0.979 ± 0.016	0.974 ± 0.009
Exodermis	0.878 ± 0.187	0.847 ± 0.084
Endodermis	0.912 ± 0.045	0.858 ± 0.046
Cortex	0.971 ± 0.040	0.982 ± 0.009
Epidermis	0.820 ± 0.131	0.738 ± 0.102
Aerenchyma	0.601 ± 0.265	0.835 ± 0.085

Note: Per-anatomical-class IoU (mean ± s.d. across samples) for RADIX on the in-distribution test set (n = 185 samples) and on the out-of-distribution set (n = 35 samples). Values correspond to Figure 2b and 2c. Aerenchyma is computed only over samples in which the class is present; Solanums lack the class entirely.

bioRxiv preprint doi: https://doi.org/10.64898/2026.08.07.743584; this version posted August 10, 2026. The copyright holder for this preprint (which was not certified by peer review) is the author/funder, who has granted bioRxiv a license to display the preprint in perpetuity. It is made available under a CC-BY 4.0 International license.

Supplementary Table 3. Per-class IoU and sample-level mIoU of RADIX, partitioned by species and by microscope.

Group	n	Vascular	Exodermis	Endodermis	Cortex	Epidermis	Aerenchyma	mIoU
By species								
Rice	92	0.973 ± 0.016	0.809 ± 0.241	0.874 ± 0.057	0.970 ± 0.045	0.742 ± 0.175	0.755 ± 0.179	0.854 ± 0.085
Millet	32	0.984 ± 0.006	0.930 ± 0.034	0.937 ± 0.021	0.972 ± 0.013	0.874 ± 0.042	0.286 ± 0.213	0.834 ± 0.045
Sorghum	59	0.987 ± 0.005	0.895 ± 0.104	0.920 ± 0.028	0.980 ± 0.041	0.835 ± 0.047	0.671 ± 0.206	0.883 ± 0.048
Solanums	37	0.974 ± 0.022	0.949 ± 0.028	0.922 ± 0.024	0.972 ± 0.018	0.864 ± 0.039	n/a	0.936 ± 0.018
By microscope								
Olympus	161	0.980 ± 0.017	0.870 ± 0.199	0.916 ± 0.044	0.970 ± 0.043	0.814 ± 0.138	0.593 ± 0.271	0.868 ± 0.078
C10	24	0.974 ± 0.010	0.930 ± 0.040	0.887 ± 0.039	0.983 ± 0.007	0.862 ± 0.046	0.675 ± 0.180	0.905 ± 0.040
Zeiss	35	0.974 ± 0.009	0.847 ± 0.084	0.858 ± 0.046	0.982 ± 0.009	0.738 ± 0.102	0.835 ± 0.085	0.872 ± 0.042

Note: Per-anatomical-class IoU and per-sample mIoU (mean ± s.d. across samples) for RADIX, broken down by species (top block) and by microscope (bottom block). Sample-level mIoU is the unweighted mean over the six anatomical structures for each sample. Rice pools both test sets (in-distribution and Zeiss out-of-distribution); the other species use the in-distribution test set only. Microscope rows are non-overlapping by construction; Zeiss is the held-out out-of-distribution row. n is the number of samples per row. Aerenchyma is computed only over samples in which the class is present; Solanums lack the class entirely. Values correspond to Figure 2d and 2e.

bioRxiv preprint doi: https://doi.org/10.64898/2026.08.07.743584; this version posted August 10, 2026. The copyright holder for this preprint (which was not certified by peer review) is the author/funder, who has granted bioRxiv a license to display the preprint in perpetuity. It is made available under a CC-BY 4.0 International license.

Supplementary Table 4. Inter-annotator IoU compared with RADIX prediction IoU, for each of 16 re-annotated samples.

			Vascular		Exodermis		Endodermis		Cortex		Epidermis		Aerenchyma		mIoU	
**Species**	**Microscope**	**Genotype**	**Model**	**Human**	**Model**	**Human**	**Model**	**Human**	**Model**	**Human**	**Model**	**Human**	**Model**	**Human**	**Model**	**Human**
Millet	Olympus	AW23	0.983	0.980	0.955	0.915	0.947	0.943	0.977	0.977	0.910	0.823	0.115	0.241	0.814	0.813
	Olympus	AW23	0.991	0.991	0.914	0.890	0.961	0.957	0.977	0.965	0.858	0.747	0.133	0.000	0.806	0.758
	C10	*psy1-17*	0.959	0.946	0.968	0.937	0.821	0.815	0.994	0.990	0.915	0.864	0.492	0.000	0.858	0.758
Rice	Olympus	*wox10-15*	0.982	0.980	0.964	0.944	0.920	0.919	0.994	0.991	0.873	0.843	0.815	0.842	0.925	0.920
	Olympus	*wox10-15*	0.982	0.983	0.059	0.582	0.930	0.928	0.831	0.981	0.248	0.552	0.578	0.536	0.605	0.761
	Olympus	Teshale	0.991	0.991	0.930	0.897	0.949	0.956	0.989	0.984	0.855	0.815	0.871	0.759	0.931	0.901
	Olympus	SRN39	0.992	0.989	0.950	0.947	0.946	0.941	0.988	0.989	0.853	0.794	0.287	0.567	0.836	0.871
Sorghum	Olympus	SRN39	0.991	0.993	0.910	0.909	0.943	0.947	0.983	0.982	0.832	0.779	0.255	0.360	0.819	0.828
	Olympus	SQR	0.991	0.994	0.966	0.938	0.943	0.958	0.994	0.990	0.910	0.861	0.765	0.703	0.928	0.907
	Olympus	Teshale	0.988	0.845	0.942	0.103	0.926	0.289	0.992	0.802	0.829	0.010	0.870	0.547	0.924	0.433
	Olympus	Teshale	0.990	0.988	0.774	0.826	0.911	0.927	0.974	0.983	0.772	0.814	n/a	n/a	0.884	0.908
	C10	M82	0.979	0.960	0.963	0.901	0.915	0.885	0.988	0.973	0.894	0.770	n/a	n/a	0.948	0.898
	Olympus	*slasft*	0.876	0.899	0.951	0.918	0.846	0.774	0.944	0.936	0.874	0.828	n/a	n/a	0.898	0.871
Solanums	Olympus	M82	0.987	0.968	0.968	0.894	0.940	0.874	0.988	0.958	0.906	0.799	n/a	n/a	0.958	0.899
	Olympus	M82	0.971	0.967	0.952	0.918	0.914	0.898	0.966	0.942	0.889	0.833	n/a	n/a	0.939	0.912
	Olympus	INS1	0.980	0.961	0.962	0.931	0.932	0.914	0.988	0.980	0.863	0.824	n/a	n/a	0.945	0.922

Note: For each of the 16 samples that were independently re-annotated by a second annotator, the table reports two IoU values per anatomical class side by side: the IoU of the RADIX prediction against the ground truth (Model) and the IoU of the second annotator against the same ground truth (Human). The rightmost mIoU pair averages each side over the six anatomical classes for that sample. n/a indicates the class is absent from the sample's ground truth (the five solanum samples and one sorghum sample lack aerenchyma).

bioRxiv preprint doi: https://doi.org/10.64898/2026.08.07.743584; this version posted August 10, 2026. The copyright holder for this preprint (which was not certified by peer review) is the author/funder, who has granted bioRxiv a license to display the preprint in perpetuity. It is made available under a CC-BY 4.0 International license.

Supplementary Table 5. Sample-level mIoU on the in-distribution and out-of-distribution test sets for the eight benchmarked architectures.

Model	Encoder	Pretrained Dataset	In-distribution Test mIoU (mean ± s.d., n = 185)	Out-of-distribution Test mIoU (mean ± s.d., n = 35)
RADIX	DINOv3-S/16	LVD-1.69B	0.873 ± 0.076	0.872 ± 0.042
DINOv2 + DPT	DINOv2-S/14	LVD-142M	0.869 ± 0.071	0.872 ± 0.034
ResNet50 + UNet++	ResNet50	ImageNet-1k	0.868 ± 0.075	0.832 ± 0.055
ResNet34 + UNet++	ResNet34	ImageNet-1k	0.867 ± 0.078	0.854 ± 0.048
YOLO26m-seg	YOLO26m	COCO	0.866 ± 0.079	0.785 ± 0.112
DINOv2 + MS-Linear	DINOv2-S/14	LVD-142M	0.858 ± 0.077	0.846 ± 0.036
SegDINO-MLP	DINOv3-S/16	LVD-1.69B	0.856 ± 0.071	0.836 ± 0.042
MicroSAM + UNETR	SAM ViT-B	SA-1B + LM	0.827 ± 0.088	0.811 ± 0.059

Note: Sample-level mIoU (mean ± s.d. across samples) for every model in the Figure 2f, evaluated on the in-distribution test set (n = 185) and the Zeiss out-of-distribution set (n = 35). Sample-level mIoU is the unweighted mean over the six anatomical classes for each sample. The Encoder and Pretrained Dataset columns identify the encoder backbone and the dataset its weights were pretrained on. RADIX shows the highest IoU score on both in-distribution and out-of-distribution test sets.

bioRxiv preprint doi: https://doi.org/10.64898/2026.08.07.743584; this version posted August 10, 2026. The copyright holder for this preprint (which was not certified by peer review) is the author/funder, who has granted bioRxiv a license to display the preprint in perpetuity. It is made available under a CC-BY 4.0 International license.

Supplementary Table 6. Ethiopian Sorghum germplasm

ID	Genotype	Variety	Region/Origin
1	Wetetbegunchie	Local land race	Wollo
11	Bobered	Local Land Race	Benishangoul Gumuz
17	Framida	Striga-resistant	Purdue University
23	Teshale	Preferred Ethiopian Farmer Variety	Melkassa Agricultural Research Center
37	Jigurti	High Striga Germination Stimulant/Land Race	Jigurti
42	ETWS 91323	Drought Tolerant, Low Germination Stimulant	Mondi/Menesibu
45	IS 38343	Drought Tolerant	Oromia/Amhara
50	ETSL 100726	Drought Tolerant	Melkassa Agricultural Research Center
51	ETSL 100576	Drought Tolerant	Melkassa Agricultural Research Center
56	10 SR-RIBKA = 235921	Sweet/Grain Sorghum	Tigray

bioRxiv preprint doi: https://doi.org/10.64898/2026.08.07.743584; this version posted August 10, 2026. The copyright holder for this preprint (which was not certified by peer review) is the author/funder, who has granted bioRxiv a license to display the preprint in perpetuity. It is made available under a CC-BY 4.0 International license.

Supplementary Table 7. Ablation study for training and loss function.

**Variant**	**In-distribution Test mIoU (mean ± s.d., n = 185)**	**Out-of-distribution Test mIoU (mean ± s.d., n = 35)**
Encoder training strategy		
Frozen encoder	0.881 ± 0.066	0.881 ± 0.034
Fine-tuned encoder (RADIX)	0.889 ± 0.065	0.888 ± 0.036
Loss function		
Dice + Focal + weighted CE	0.885 ± 0.064	0.879 ± 0.038
Dice + Focal + weighted CE + Lovasz (RADIX)	0.889 ± 0.065	0.888 ± 0.036

bioRxiv preprint doi: https://doi.org/10.64898/2026.08.07.743584; this version posted August 10, 2026. The copyright holder for this preprint (which was not certified by peer review) is the author/funder, who has granted bioRxiv a license to display the preprint in perpetuity. It is made available under a CC-BY 4.0 International license.

Supplementary Data

Supplementary Data 1. Training and validation set diversity (n = 1293 train, 182 val).

Species	Genotype	Train	Val
Millet	AW23	71	7
	Kitaake	86	52
	psy1-17	185	0
Rice	*psy1-9*	105	3
	wox10-15	33	0
	wox10-50	31	0
	2005MI5064	0	3
	235464	4	0
	235467	3	0
	235761	4	0
	235763	3	0
	235921	4	0
	235925	4	0
	A2267-2	6	0
	B35	0	1
	Dekeba	4	0
	ETSL 100016	9	0
	ETSL 100046	3	0
	ETSL 100143	4	0
	ETSL 100305	0	2
	ETSL 100307	3	0
Sorghum	ETSL 100308	5	0
	ETSL 100309	4	0
	ETSL 100312	3	0
	ETSL 100316	4	0
	ETSL 100726	4	0
	ETSL 101295	3	0
	ETSL 101728	6	0
	ETSL 101843	0	2
	ETSL 101844	5	0
	ETSL 101845	5	0
	ETSL 101847	5	0
	ETSL 101851	4	0
	ETSL 101853	0	2
	ETSL 101857	0	3
	ETSL 101862	4	0
	ETSL 101865	7	0
	ETSL 101866	4	0
	ETSL 101888	3	0
	ETWS 90754	3	0
	ETWS 90787	3	0
	ETWS 91242	3	0
	ETWS 91323	4	0
	Framida	5	0
	Gobiye	5	0
	IESV 23005DL	0	1
	IS 38306	4	0
Sorghum	IS 38343	6	0
	IS 38408	5	0
	Macia	4	0
	Melkam	5	0
	Misikir	0	2
	NTJ2	0	2
	P9501B	0	2
	S35	5	0
	SQR	16	9
	SRN	9	2
	Teshale	151	18
	cerasiforme	0	2
	LA0513	0	7
Solanum lycopersicum	M82	301	19
	slasft	49	0
	slmyb92	66	21
	VF36	0	3
Solanum aethiopicum	PI424860	9	0
Solanum anguivi	ANG1	0	1
Solanum arcanum	LA2157	0	2
Solanum candidum	994750097	7	0
Solanum cheesmaniae	LA1407	3	0
Solanum habrochaites	LA1777	0	2
Solanum huaylasense	LA1364	0	2
Solanum insanum	INS1	0	5
Solanum peruvianum	LA1777	2	0
Solanum pimpinellifolium	LA1589	0	3
Solanum quitoense	PI489701	0	3
Solanum violaceum	PI319855	0	1

bioRxiv preprint doi: https://doi.org/10.64898/2026.08.07.743584; this version posted August 10, 2026. The copyright holder for this preprint (which was not certified by peer review) is the author/funder, who has granted bioRxiv a license to display the preprint in perpetuity. It is made available under a CC-BY 4.0 International license.

Supplementary Data 2. Imaging metadata

[Attached as an .xlsx file]

Supplementary Data 3. Annotation SOP

[Attached as an .docx file]

bioRxiv preprint doi: https://doi.org/10.64898/2026.08.07.743584; this version posted August 10, 2026. The copyright holder for this preprint (which was not certified by peer review) is the author/funder, who has granted bioRxiv a license to display the preprint in perpetuity. It is made available under a CC-BY 4.0 International license.

SOP: Fluorescence Microscopy Image Annotation for Plant Root Segmentation

Authors: Stefan Sanow, Yifei Gu, Tamera Taylor, Kevin Morimoto

Email: ssanow@ucdavis.edu · bmegu@ucdavis.edu

1. Purpose

This SOP provides standardized guidelines for generating consistent and accurate segmentation masks for tissue regions in fluorescence microscopy images of plant root cross-sections. These annotations are used to train and validate machine learning models that automatically identify and quantify key root structures. Annotations must be precise, as structural areas are measured in pixels.

2. Root Cross-Section Anatomy

Common Structures (All Species)

Epidermis: The outermost layer of the root, forming the interface between root tissue and the surrounding background. It appears as a layer of connected cells encircling the entire cross-section. The epidermis boundary defines the outer limit of the 'whole root' annotation. In dicots (tomato), root hair extensions may be present and should be excluded from annotations.

Exodermis: The second outermost cell layer, lying directly beneath the epidermis and encasing the cortex. Exodermal cells are larger and more polygonal than epidermal cells. In multichannel images, the exodermis typically shows higher signal in two of three channels. It is annotated as two concentric rings: the outer exodermis (epidermis-facing boundary) and inner exodermis (cortex-facing boundary).

Endodermis: A thin layer of cells separating the cortex from the vascular cylinder. In dicots, it is the second full concentric layer from the vascular center (pericycle is first) and can be identified by distinct dot structures and banding patterns in multichannel images. It is annotated as two concentric rings: the outer endodermis (cortex-facing boundary) and inner endodermis (pericycle-facing boundary).

Monocot-Specific Structure (Millet, Rice, Sorghum)

Aerenchyma: Air-filled cavities that form within the cortex, appearing as dark, irregular holes between cells. They vary in size and shape. Not all monocot root images contain aerenchyma; roots without aerenchyma are annotated with zero polygons for label 1.

3. Annotation Classes

All species share the same 6-class label schema (indices 0–5). Classes that are biologically absent in a given species are annotated with zero polygons; this is real biology, not missing data.

bioRxiv preprint doi: https://doi.org/10.64898/2026.08.07.743584; this version posted August 10, 2026. The copyright holder for this preprint (which was not certified by peer review) is the author/funder, who has granted bioRxiv a license to display the preprint in perpetuity. It is made available under a CC-BY 4.0 International license.

Label Index	Label	Description	Approximate Location / Notes
0	Whole Root	Outermost boundary of the root, separating sample from background.	Trace along the epidermis (outermost visible cell wall), ensuring the outermost cell wall is contained within the annotation mask. All species.
1	*Aerenchyma*	*Air spaces or cavities within the cortex.*	*Appear as dark, irregular holes in the cortex. Monocots (millet, rice, sorghum) only; annotate zero polygons in dicots.*
2	Outer Endodermis	Outer edge of the endodermis.	Trace the boundary between cortex and endodermis; contain the endodermis cell wall within the annotation mask. All species.
3	Inner Endodermis	Inner edge of the endodermis.	Trace the boundary between endodermis and pericycle; exclude the endodermis cell wall from the masked area. All species.
4	Outer Exodermis	Outer edge of the exodermis.	Trace the boundary between epidermis and exodermis; contain the exodermis cell wall within the annotation mask. All species.
5	Inner Exodermis	Inner edge of the exodermis.	Trace the boundary between exodermis and cortex; exclude the exodermis cell wall from the annotation mask. All species.

Shaded row: biologically absent in dicots (zero polygons expected).

4. Annotation Guidelines

A. General Rules

1. Always follow visible cell walls when outlining boundaries.
2. Keep polygons clean, smooth, and non-overlapping.
3. When zooming in to annotate, periodically zoom out to ensure consistency across the full root section.

B. Whole Root Boundary

- This is the outermost contour of the root cross-section.
- Follow the epidermal layer tightly; do not include the background.
- Ignore detached debris, root hairs (dicots), or broken edges outside the main root structure.
- If a lateral root disrupts the boundary, draw across it to ensure a continuous closed polygon.

bioRxiv preprint doi: https://doi.org/10.64898/2026.08.07.743584; this version posted August 10, 2026. The copyright holder for this preprint (which was not certified by peer review) is the author/funder, who has granted bioRxiv a license to display the preprint in perpetuity. It is made available under a CC-BY 4.0 International license.

C. Endodermis

- The outer and inner endodermis boundaries form concentric rings enclosing a narrow band of specialized cells.
- **Outer endodermis (label 2):** annotate at the pixels just toward the cortex-facing side of the endodermal cells.
- **Inner endodermis (label 3):** annotate at the pixels just toward the pericycle-facing side of the endodermal cells.
- Maintain continuous, ring-like boundaries. If the line is dim, infer its likely continuation to complete the ring smoothly.
- If a lateral root disrupts the endodermis, draw across it to keep both rings continuous.

D. Exodermis

- The outer and inner exodermis boundaries form concentric rings enclosing the layer of cells directly beneath the epidermis.
- **Outer exodermis (label 4):** annotate at the pixels just toward the epidermis-facing side of the exodermal cells.
- **Inner exodermis (label 5):** annotate at the pixels just toward the cortex-facing side of the exodermal cells.
- Maintain continuous, ring-like boundaries. If a line is dim, infer its likely continuation to complete the ring smoothly.
- If a lateral root disrupts the exodermis, draw across it to keep both rings continuous.

E. Aerenchyma - Monocots only (label 1)

- **Aerenchyma only occurs in the cortex.** Do not annotate gaps in the endodermis or epidermis — these are likely mechanical damage.
- Annotate each distinct cortical cavity as one polygon, placed just inside the cellular boundaries.
- Annotate small cavities individually if their shape is clearly defined.
- A single polygon should not cover more than 25% of the total cortex area, unless the aerenchyma is very large and continuous.
- If unsure whether a cavity is aerenchyma, annotate it — it is easier to remove uncertain annotations than to add missing ones.
- Some images contain no aerenchyma; leave label 1 with zero polygons for those images.

5. Quality Control Checklist

Before finalizing annotations, verify that:

- All 6 label types are present and correctly indexed (0–5); biologically absent classes have zero polygons.
- All boundaries are smooth, closed polygons.
- No overlapping regions exist between classes.

bioRxiv preprint doi: https://doi.org/10.64898/2026.08.07.743584; this version posted August 10, 2026. The copyright holder for this preprint (which was not certified by peer review) is the author/funder, who has granted bioRxiv a license to display the preprint in perpetuity. It is made available under a CC-BY 4.0 International license.

- Aerenchyma annotations (label 1) are confined to the cortex in monocots; zero polygons in dicots.
- The root edge (label 0) follows the true epidermal boundary.
- Regions with ambiguous gaps or broken cells have been double-checked.

6. Annotation Format

Export annotations in **YOLO-style segmentation format** as .txt files. Each line contains the label index followed by normalized x,y coordinate pairs defining the polygon vertices:

```
<label_index>  x1 y1  x2 y2  x3 y3  ...
```

Example (label 2 — Outer Endodermis):

```
2  0.399595 0.479795  0.402324 0.477905  0.405264 0.476646  0.407783 0.474546  ...
```
